# Arterial pH, bicarbonate levels and base deficit at presentation as markers of predicting mortality in acute pancreatitis: a single-centre prospective study

**DOI:** 10.1093/gastro/gou037

**Published:** 2014-07-03

**Authors:** Vishal Sharma, Thingbaijam Shanti Devi, Ravi Sharma, Puneet Chhabra, Rajesh Gupta, Surinder S. Rana, Deepak K. Bhasin

**Affiliations:** ^1^Department of Gastroenterology, Post Graduate Institute of Medical Education and Research, Chandigarh, India and ^2^Department of Surgery, Post Graduate Institute of Medical Education and Research, Chandigarh, India

**Keywords:** acute pancreatitis, base deficit, MA, organ failure

## Abstract

**Background.** Arterial blood gas (ABG) parameters such as pH form part of multi-parameter scoring systems for predicting severe acute pancreatitis; however, literature on detailed evaluation of ABG alone in this context is scarce.

**Methods.** Patients with acute pancreatitis presenting to our unit between January 2012 and November 2013 were prospectively studied. ABG analysis was done at admission and development of organ failure, any need for intervention, and mortality were noted. The association between various parameters of ABG analysis and the development of organ failure or local complications, need for interventions (endoscopic/radiological/surgical) and mortality were analysed.

**Results.** Two hundred and five patients (mean age: 39.33 ± 13.85 years; 61.0% males) were prospectively studied. The aetiology of acute pancreatitis was alcohol in 93 patients (45.4%) and gall stone disease in 73 (35.6%). Organ failure developed in 71.2% patients and 83.9% had local complications. In 18% of patients, endoscopic/radiological/surgical interventions were needed and 14.6% died. The patients (*n = *35) with metabolic acidosis (pH <7.35) suffered higher frequency of organ failure, need for interventions and mortality. Patients with low arterial bicarbonate levels, as well as higher base deficit, also displayed higher frequency of organ failure, need for interventions and mortality. The receiver operating characteristic (ROC) curves for pH <7.35, bicarbonate <22 meq/L and base deficit of >−4 meq/L for prediction of mortality were 0.771 (95% CI: 0.664–0.878), 0.707 (95% CI: 0.622–0.791) and 0.780 (95% CI: 0.693–0.867), respectively.

**Conclusion.** Arterial pH, bicarbonate levels, and base deficit at presentation are useful early markers for predicting adverse outcome in acute pancreatitis.

## INTRODUCTION

Acute pancreatitis (AP) is an acute inflammatory process of the pancreas that can result in local or systemic complications [1]. The assessment of severity of AP is one of the most important issues in its management. Although multiple criteria are available for prognosis and determination of severity of AP, there is a need to identify better predictors of outcome. Various prognostic factors and scoring systems have been proposed for accurate assessment of the severity of AP and reliable prediction of high-risk and potentially fatal cases; some of them include multiple biochemical parameters and therefore result in a cumbersome scoring, as in the Ranson’s criteria, while others have attempted to simplify the prediction of severity, as in the Bed Side Index for Severity of Acute Pancreatitis (BISAP) scoring system [2, 3]. With respect to scoring systems, the most widely validated of these remains the Acute Physiology and Chronic Health Examination (APACHE) II score [4]. These all have comparable levels of overall accuracy.

The development of metabolic acidosis (MA) is a common occurrence during critical illness. Early and accurate identification and correction of significant MA is particularly relevant to patients in the intensive care unit (ICU). Arterial base deficit/excess (BD/E) is a commonly used marker of MA in critically ill patients. MA has been one of the criteria used for predicting a severe course of AP; it is one of the factors measured within 48 hours of admission as a part of Ranson’s scoring system [2]. Arterial pH is also a component of the APACHE-II scoring system that is commonly used in AP [5]. MA can occur in AP for multiple reasons that include lactic acidosis resulting from shock, renal failure or—late in the course of disease—because of loss of bicarbonate-rich pancreatic secretions due to pancreatic duct disruption. There is paucity of data on the prognostic value of various individual parameters of arterial blood gas (ABG) analysis carried out in AP patients at admission. The objective of the present study was to evaluate arterial pH, bicarbonate levels and base deficit at admission in patients with AP and to study their value in predicting organ failure, local complications, need for surgery or intervention and mortality.

## PATIENTS AND METHODS

All consecutive patients with a diagnosis of AP presenting to our unit—in a large tertiary care hospital in North India—were prospectively enrolled. The diagnosis of AP was based on suggestive clinical history (acute onset upper abdominal pain—with or without radiation to back—vomiting, constipation and obstipation), physical examination, and elevated serum amylase to greater than three times the upper limit of normal and/or positive abdominal imaging [1]. Informed consent was obtained from all the patients prior to enrolment in the study and they were given standard medical care throughout the study period. Those with coronary artery disease/pre-existing cardiac disorder, diabetes mellitus, renal failure, malignancy, pregnancy, chronic obstructive pulmonary disease, underlying chronic pancreatitis, pancreatic malignancy, underlying acid base disorder, who presented >10 days after the onset of pain, plus those patients refusing consent for participation, were excluded. All the participants were thoroughly interviewed and subjected to clinical and laboratory examination. Contrast-enhanced computerized tomography (CECT) of the abdomen was done 72 hours after diagnosis, to stage the severity according to the Balthazar Computed Tomography Severity Index (CTSI). The severity of AP was assessed in terms of duration of hospitalization, organ failure, local complications (fluid collections, organised necrosis), CTSI, mortality and the need for surgery/intervention. The study protocol was approved by the Institute Ethics Committee, PGIMER, Chandigarh.

Haematological investigations, blood sugar, serum amylase, blood urea, serum creatinine, albumin, globulin, calcium, phosphate, ABG analysis and liver function tests were carried out on the day of admission. For ABG analysis, 1 mL of blood was collected from the radial artery into heparinized syringes and immediately analysed using the Cobas b 121 analyser (Roche, Germany). The analysis included measurement of arterial pH, the partial pressure of arterial carbon dioxide (PaCO_2_), and the partial pressure of arterial oxygen (PaO_2_). Standard bicarbonate (HCO_3_) was calculated from the observation parameters pH and PaCO_2_. Base deficit/excess (BD/E), which represents the amount of acid or base required to normalize the pH in a litre of blood, was directly calculated by the blood gas analyser from PaCO_2_, pH and serum HCO_3_ values as applied to a standard normogram.

Patients received standard treatment (oxygen, invasive ventilation, vasopressor, dialysis, etc.) to support organ failure (if any). Cultures from blood and urine were obtained upon suspicion of infection, and intravenous antibiotics were administered to treat infection according to culture and sensitivity reports. In case of non-response, patients with infected pancreatic necrosis and/or fluid collections—with or without persistent organ failure—were subjected to intervention (image-guided percutaneous catheter (PCD), endoscopic transmural drainage, or surgery) and cultures from the drain fluid were obtained. A symptom review was carried out at regular intervals and any new findings were recorded. Patients were followed clinically throughout their admission and subjected to repeated haematological tests and abdominal ultrasound/CECT examination as when indicated. Systemic and local complications of acute pancreatitis were defined in accordance with the revised Atlanta Classification [1].

These patients were followed up to clinical recovery or death. The associations between various parameters of ABG analysis and development of organ failure or local complications, need for interventions (endoscopic/radiological/surgical) and mortality were analysed.

### Statistical analysis

The descriptive data were presented as percentages for categorical variables and mean ± standard deviation (SD) for quantitative variables The patients were categorized into subgroups based upon the arterial pH, bicarbonate levels and base deficit values (pH <7.35 and ≥7.35), bicarbonate levels <22 and ≤22 mEq/L and base deficit >−4 mEq/L and ≤−4 mEq/L, respectively). The continuous variables were compared using a student's *t*-test, whereas the categorical variables were compared using the Chi-squared test. The ability of various ABG parameters to predict mortality was explored using receiver operator characteristic (ROC) curves and the area under ROC curves (AUROC).

## RESULTS

Two hundred and eighty-seven AP patients were seen during the study period and 82 were excluded (19 refused consent and 63 presented to our hospital >10 days after the onset of pain). Out of 205 patients enrolled, 125 (61.0%) were males and 80 (39.0%) were females. The mean age of the patients included was 39.33 ± 13.85 years (range: 13–75 years). The aetiology of acute pancreatitis was alcohol in 93 patients (45.4%), gall stone disease in 73 (35.6%), idiopathic in 16 (7.8%) and others in 23 patients (11.2%). The other aetiologies were mixed in 8, trauma in 5, hypertriglyceridemia in 5, post-viral in 3 and drug-induced in 2. The mean period from onset of symptoms to presentation at our hospital was 4.24 ± 2.68 days (Table 1).

**Table 1.** Demographic profile of the patients

Mean Age	39.33 ± 13.85 years
Gender	125 males (61.0%); 80 females (39.0%).
Aetiology	Alcohol: 93 patients (45.4%)
	Gall stone disease: 73 (35.6%)
	Idiopathic: 16 (7.8%)
	Others: 23 (11.2%)
Mean duration of presentation	4.24 ± 2.68 days

One hundred and forty-six patients (71.2%) developed persistent organ failure, with multiple organ failure developing in 37 (18.0%). Respiratory failure developed in 138 (67.3%) and renal failure in 30 (14.6%), while 26 patients (12.7%) developed shock. Acute fluid collections (AFC) developed in 172 (83.9%) and interventions including percutaneous and endoscopic interventions (endoscopic transmural drainage for walled-off pancreatic necrosis), as well as surgery, were needed in 37 cases (18.0%). Thirty patients (14.6%) succumbed to their illness: early deaths (mortality within 14 days of admission) occurred in 14 (46.6%) and 16 (53.4%) died after 14 days or more.

### ABG parameters

The mean arterial pH was 7.40 ± 0.06 and pH <7.35 was present in 35 patients (17.1%). The mean arterial bicarbonate level was 22.18 ± 17.82 mEq/L, with 119 patients (58%) having values <22 mEq/L. Base deficit more than −4 mEq/L was found in 73 (35.6%) patients.

### Association of arterial pH with various outcome parameters

The patients with evidence of pH <7.35 had a higher frequency of OF (pulmonary failure, shock and renal failure), multiple organ failure, and greater need for intervention. Thirty two (91.4%) of the 35 patients with pH <7.35 experienced OF, whereas OF was present in 114/170 (67.1%) in those with pH ≥7.35 (*P = *0.004). Multiple organ failure was present in 37 patients, 13 of whom had pH <7.35 (*P = *0.003). Out of 26 patients in whom shock was present, 12 patients had arterial pH <7.35. Interventions were required in 37 patients (18.0%). Thirteen patients (37.1%) from the group with pH <7.35 needed intervention, compared with 24 (14.1%) from the other group (*P* = 0.003). Nineteen patients with arterial pH <7.35 succumbed to their illness, whereas 11 patients with pH value ≥7.35 died, and this difference was statistically significant (*P* = 0.000) (Table 2).

**Table 2.** Association of arterial pH with various outcome parameters

**Variable**	**pH <7.35** (*n = *35) *n* (%)	**pH ≥7.35** (*n = *170) *n* (%)	***P*-value**
Acute fluid collection	30 (85.7)	142 (83.5)	0.793
Organ failure	32 (91.4)	114 (67.1)	**0.004**
Multiple organ failure	13 (37.1)	24 (14.1)	**0.003**
Shock	12 (34.3)	14 (8.2)	**0.000**
Respiratory failure	30 (85.7)	108 (63.5)	**0.010**
Renal failure	16 (45.7)	14 (8.2)	**0.000**
Intervention	13 (37.14)	24 (14.1)	**0.003**
Mortality	19 (54.3)	11 (6.5)	**0.000**

### Association of arterial HCO_3_ with various outcome parameters

We compared outcomes in the two bicarbonate groups, i.e those with arterial HCO_3_ of less than 22 *vs* those ≥22 mEq/L. Ninety-six (80.7%) of the 119 patients with bicarbonate <22 mEq/L had OF, whereas OF was present in 50 (58.1%) in those with bicarbonate levels 22 mEq/L (*P* = 0.001). MOF was present in 37 patients, 31 of whom had bicarbonate <22 mEq/L (*P* < 0.001). Twenty-one (80.8%) of twenty-six patients with shock had bicarbonate <22 mEq/L (*P* = 0.018). Respiratory failure was present in 138 patients and 89 of these had bicarbonate levels <22 mEq/L. Of the 30 patients who had renal failure, 28 had bicarbonate levels <22 mEq/L. Intervention was required in 29 out of 119 patients with bicarbonate <22 mEq/L (24.4%) and this was significantly higher (*P* = 0.006) than the frequency of interventions in patients with normal bicarbonate [8 patients (9%)]. Out of 30 deaths in this study, 28 (93.3%) had bicarbonate <22 mEq/L. The mortality was significantly higher in patients with low bicarbonate levels (*P* < 0.001) (Table 3).

**Table 3.** Association of arterial HCO_3_ with various outcome parameters

**Variable**	**HCO_3_ ≤22** (*n = *119) *n* (%)	**HCO_3_ >22** (*n = *86) *n* (%)	***P-*value**
Acute fluid collection	100 (84.0)	72 (83.7)	0.839
Organ failure	96 (80.7)	50 (58.1)	**0.001**
Multiple organ failure	31 (26.1)	6 (6.9)	**0.000**
Shock	21 (17.6)	5 (5.8)	**0.018**
Respiratory failure	89 (74.8)	49 (56.9)	**0.010**
Renal failure	28 (23.5)	2 (2.3)	**0.000**
Intervention	29 (24.4)	8 (9.3)	**0.006**
Mortality	28 (23.5)	2 (2.3)	**0.000**

### Association of arterial base deficit with various outcome parameters

Seventy-three patients had base deficit of >−4, whereas 132 had base deficit of a-4 mEq/L. Organ failure was present in 60 of the patients (82.2%) with base deficit >−4 mEq/L compared with 86 (65.2%) in the other group (*P* = 0.010). MOF was seen in 37 patients and 24 of these had base deficit >−4 mEq/L. Of the 26 patients with shock, 19 (73.1%) had base deficit >−4 mEq/L. Respiratory failure was present in 54 (73.9%) patients with base deficit >−4 meq/L and was present in 84 (63.6%) patients in the other group; the difference between the two groups was not significant (*P* = 0.162). The frequencies of renal failure (31.5% *vs* 5.3%) and shock (23.3% and 6.8%) were higher in the greater-base-deficit group. Seventeen (23.3%) patients with BD >−4 mEq/L required intervention, as compared with 20 (15.2%) in patients with lower base deficit and the difference did not reach statistical significance. Twenty-five deaths in this study (34.2%) occurred in patients with base deficit >−4 mEq/L, compared with 5 deaths (3.8%) in the second group. The mortality was significantly higher in these patients (*P* = 0.000) (Table 4).

**Table 4.** Association of arterial base deficit with various outcome parameters

**Variable**	**Base Deficit >−4.0** (*n = *73) *n* (%)	**pH <−4.0** (*n = *132) *n* (%)	***P-*value**
Acute fluid collection	61 (83.6)	111 (80.1)	1.000
Organ failure	60 (82.2)	86 (65.2)	**0.010**
Multiple organ failure	24 (32.9)	13 (9.8)	**0.000**
Shock	17 (23.3)	9 (6.8)	**0.002**
Respiratory failure	54 (73.9)	84 (63.6)	0.162
Renal failure	23 (31.5)	7 (5.3)	**0.000**
Intervention	17 (23.3)	20 (15.2)	0.184
Mortality	25 (34.2)	5 (3.8)	**0.000**

**Figure 1.**
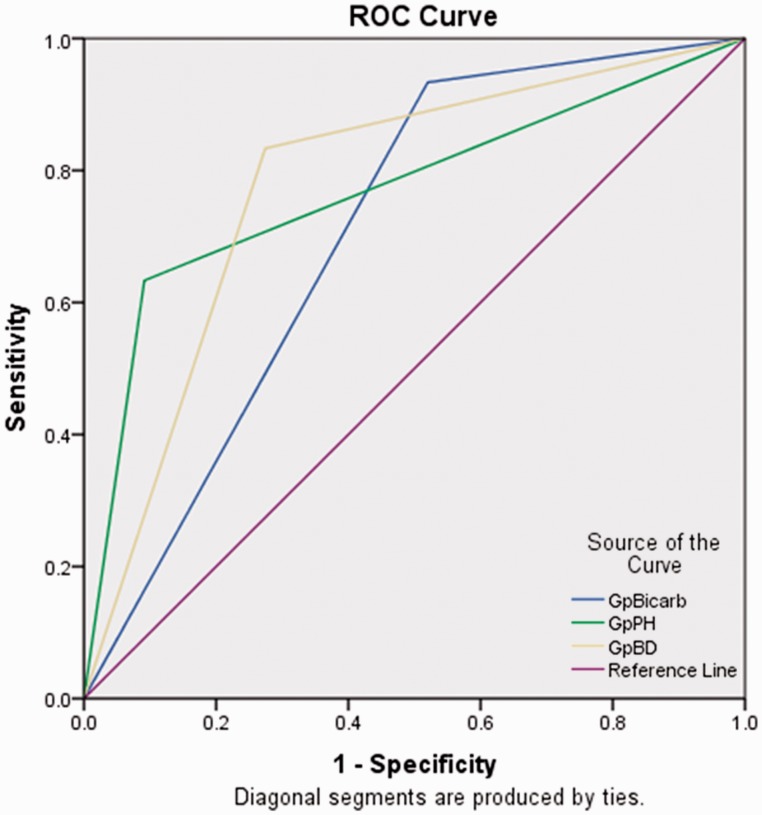
The receiver operating curves (ROC) of arterial pH, bicarbonate levels and base deficit in predicting mortality.

The area under the ROC curve for pH <7.35, bicarbonate < 22 meq/L and base deficit of >−4 meq/L for prediction of mortality was 0.771 (95% CI: 0.664–0.878), 0.707 (95% CI: 0.622–0.791) and 0.780 (95% CI: 0.693–0.867), respectively (Figure 1).

## DISCUSSION

Predicting the severity and outcome of AP remains challenging for a physician. Although multiple scoring systems, as well as single markers, are being used for predicting a severe outcome in AP, there is no consensus regarding the use of one or the other in clinical practice. Arterial blood gas parameters have not been evaluated for prediction of severity and outcome in acute pancreatitis, even though individual parameters form components of multi-score assessment like Ranson’s and APACHE [2, 3]; in fact one of the reports criticised ABG as an unnecessary tool in AP patients. This report, unfortunately, focused primarily on the blood gas- and oxygenation-related parameters [6]. In other critical illnesses there is sufficient evidence to implicate ABG parameters as important predictors of outcome.

It has been observed that patients with organ failure at admission have higher mortality than those who do not [7–9]. The development of organ failure, whether at admission or thereafter, implies a worse prognosis. The highest mortalities are among patients with multiple- and persistent organ failure [8, 9]. In the present series, 146 patients (71.2%) suffered organ failure, which was present in 32 of the 35 patients (91.4%) with pH levels <7.35 and this frequency was significantly higher than the patients with pH >7.35 (67.1%). Also, 80.7% patients with bicarbonate <22 mEq/L experienced organ failure, in contrast with 58.1% patients with a higher bicarbonate level (*P = *0.001). Sixty of seventy-three patients (82.2%) with a higher base deficit suffered organ failure, which was significantly more than the group with lower base deficit (65.2%). The association between acidosis and an increase in multiple organ failure and mortality for intensive care patients has long been known.

In the present study Group, interventions were required in 37 patients (18.05%). Thirteen (37.14%) with pH <7.35 underwent interventions, as compared with 14.1% with pH 7.35. The need for intervention was significantly more in patients with arterial pH <7.35 and patients with lower bicarbonate. Studies of factors at admission, that predict the need for intervention, are scarce. A meta-analysis by the Dutch Pancreatitis Study Group, which included 384 patients, reported that the presence of organ failure and infected pancreatic necrosis could predict the need for intervention [10]. A subset of patients among those managed by the step-up approach will still require surgery. A prospective study included 70 consecutive patients with severe AP to identify factors that could predict surgical intervention after initial management with PCD. Reversal of sepsis within a week of PCD, APACHE II score at first intervention, and organ failure within a week of the onset of disease, could predict the need for surgery in the early course of disease. The number of patients with MA (pH <7.2) and base deficit >5 mEq/L at admission was significantly higher in the pancreatic necrosectomy group, compared with the PCD-alone group, and may predict failure of PCD [11].

Nineteen out of our 35 patients (54.3%) with pH <7.35 died and 28 patients (23.5%) with bicarbonate <22 mEq/L and 25 patients (24.2%) with base deficit >−4 mEq/L also succumbed to their illness. Studies in intensive care settings have documented the effects of MA on mortality. MA (bicarbonate level of less than 18 mEq/L) was seen in 23 out of 71 AP patients (32%) in a Brazilian study [12]. A study of 107 patients admitted to intensive care for various reasons demonstrated a significantly severe MA in non-survivors [13]. Another study reported that severe metabolic or mixed academia—defined by plasma pH lower than 7.20—occurs within the first 24 hours in the ICU in 6% of critically ill patients, and was associated with high rates of mortality in patients admitted to ICU [13, 14]. In a prospective, observational study of a large cohort of 530 patients, pH at presentation predicted not only long-term mortality but also ICU admission, in-hospital mortality, and mortality after 30-day follow-up [15]. Patients with a pH level of ≤7.39 showed a mortality rate of 37% after 12 months. The association of pH and mortality was independent of other predictors, both in patients with pulmonary- and other causes of acute dyspnoea.

Although many parameters—including serum creatinine, haematocrit, obesity, blood urea nitrogen, C-reactive protein, serum procalcitonin and D-dimer—have been studied previously, the present study is important because a detailed evaluation of ABG in acute pancreatitis has not previously been reported [16]. Our results suggest that pH, low bicarbonate and higher base deficit at presentation predicts an adverse outcome and worse prognosis in patients with acute pancreatitis, including the occurrence of organ failure, need for intervention and mortality. The AUROC suggests that the performances of each of these parameters are similar for prediction of mortality. Therefore, carrying out an arterial blood gas analysis in patients with acute pancreatitis not only helps in management of patients but may predict the outcome.

## CONCLUSION

In patients with acute pancreatitis, low arterial pH and bicarbonate levels and higher base deficit at presentation predict an adverse outcome with higher frequency of organ failure, need for intervention and mortality. Thus a simple diagnostic analysis of arterial blood gas in patients with acute pancreatitis can help in predicting adverse outcome.

### Authors' contributions:

1. VS: Analysis and interpretation of the data; drafting of the article
2. ST: Collection of data
3. RS: Collection of data
4. PC: Collection of data
5. RG: Provision of study materials or patients
6. SSR: Analysis and interpretation of the data; drafting of the article
7. DKB: Analysis and interpretation of the data; drafting of the article; critical revision of the article for important intellectual content

**Conflict of interest:** none declared.
